# Identification of Two Missense Mutations of *ERCC6* in Three Chinese Sisters with Cockayne Syndrome by Whole Exome Sequencing

**DOI:** 10.1371/journal.pone.0113914

**Published:** 2014-12-02

**Authors:** Shanshan Yu, Liyuan Chen, Lili Ye, Lingna Fei, Wei Tang, Yujiao Tian, Qian Geng, Xin Yi, Jiansheng Xie

**Affiliations:** 1 BGI-shenzhen, Shenzhen, 518083, China; 2 Prenatal Diagnosis Center, Shenzhen Maternity and Child Healthcare Hospital, Shenzhen, 518048, China; Pasteur Institute of Lille, France

## Abstract

Cockayne syndrome (CS) is a rare autosomal recessive disorder, the primary manifestations of which are poor growth and neurologic abnormality. Mutations of the *ERCC6* and *ERCC8* genes are the predominant cause of Cockayne syndrome, and the *ERCC6* gene mutation is present in approximately 65% of cases. The present report describes a case of Cockayne syndrome in a Chinese family, with the patients carrying two missense mutations (c.1595A>G, p.Asp532Gly and c.1607T>G, p.Leu536Trp) in the *ERCC6* gene in an apparently compound heterozygote status, especially, p.Asp532Gly has never been reported. The compound heterozygote mutation was found in three patients in the family using whole exome sequencing. The patients’ father and mother carried a heterozygous allele at different locations of the *ERCC6* gene, which was confirmed by Sanger DNA sequencing. The two mutations are both located in the highly conserved motif I of ATP-binding helicase and are considered “Damaging,” “Probably Damaging,” “Disease Causing,” and “Conserved”, indicating the role of DNA damage in the pathogenetic process of the disease. The results not only enrich the *ERCC6* mutations database, but also indicate that whole exome sequencing will be a powerful tool for discovering the disease causing mutations in clinical diagnosis.

## Introduction

Cockayne syndrome (CS; MIM 216400, 133540) is a rare autosomal recessive neurodegenerative disorder characterized by progressive growth failure, microcephaly, mental retardation, retinal pigmentary degeneration, deafness, photosensitivity, accelerated systemic degeneration of somatic tissue and premature death. Cockayne syndrome spans a phenotypic spectrum that includes CS type I, CS type II, CS type III, and Xeroderma pigmentosum-Cockayne syndrome (XP-CS). Due to the wide clinical variability, the diagnosis of this disorder is quite difficult during early stages. Molecular genetic testing can confirm the diagnosis. Mutations in the *ERCC6* and *ERCC8* genes are known to cause Cockayne syndrome [1]–[4] and approximately 65% individuals with CS have mutations in the *ERCC6* gene [5].

The *ERCC6* gene is located on chromosome 10q11.23, encoding a protein belonging to the SWI2/SNF2 family that contains an acidic domain, a glycine-rich region, two putative nuclear localized signal sequences, and seven characteristic helicase ATPase domains. It is part of the nucleotide excision repair (NER) pathway, a complex system that eliminates a broad spectrum of structural DNA lesions, including ultraviolet (UV)-induced cyclobutane pyrimidine dimers, bulky chemical adducts, and DNA cross-links. One of the NER pathways preferentially repairs lesions on the transcribed strand of active genes; this process occurs more rapidly than repairs on nontranscribed strands, which is part of overall genome repair [6].

The present study reports two *ERCC6* mutations identified by whole exome sequencing in three individuals with Cockayne syndrome from a Chinese family. The two missense mutations are c.1595A>G and c.1607T>G, which are located in exon 7 of the *ERCC6* gene, and the c.1595A>G has never been reported.

## Materials and Methods

### Samples collection

We collected a 5 ml peripheral blood sample from each person in the family.

### Ethics statement

The study was conducted in accordance with the guiding principles of the Declaration of Helsinki. Collection of samples was approved by the ethical committees of Shenzhen Maternity and Child Healthcare Hospital, and informed written consent was obtained from the parents of the three affected sisters because the sisters did not have the capacity to understand and sign written informed consent. The parents completely understood that their peripheral blood samples would be used in this research and requested genetic counseling. We followed all regulations for the enrollment of patients, sample collection and informed consent for the purpose of research. All studies using human materials in this article were approved by the ethical committee of Shenzhen Maternity and Child Healthcare Hospital.

### Clinical report

**Figure 1:**
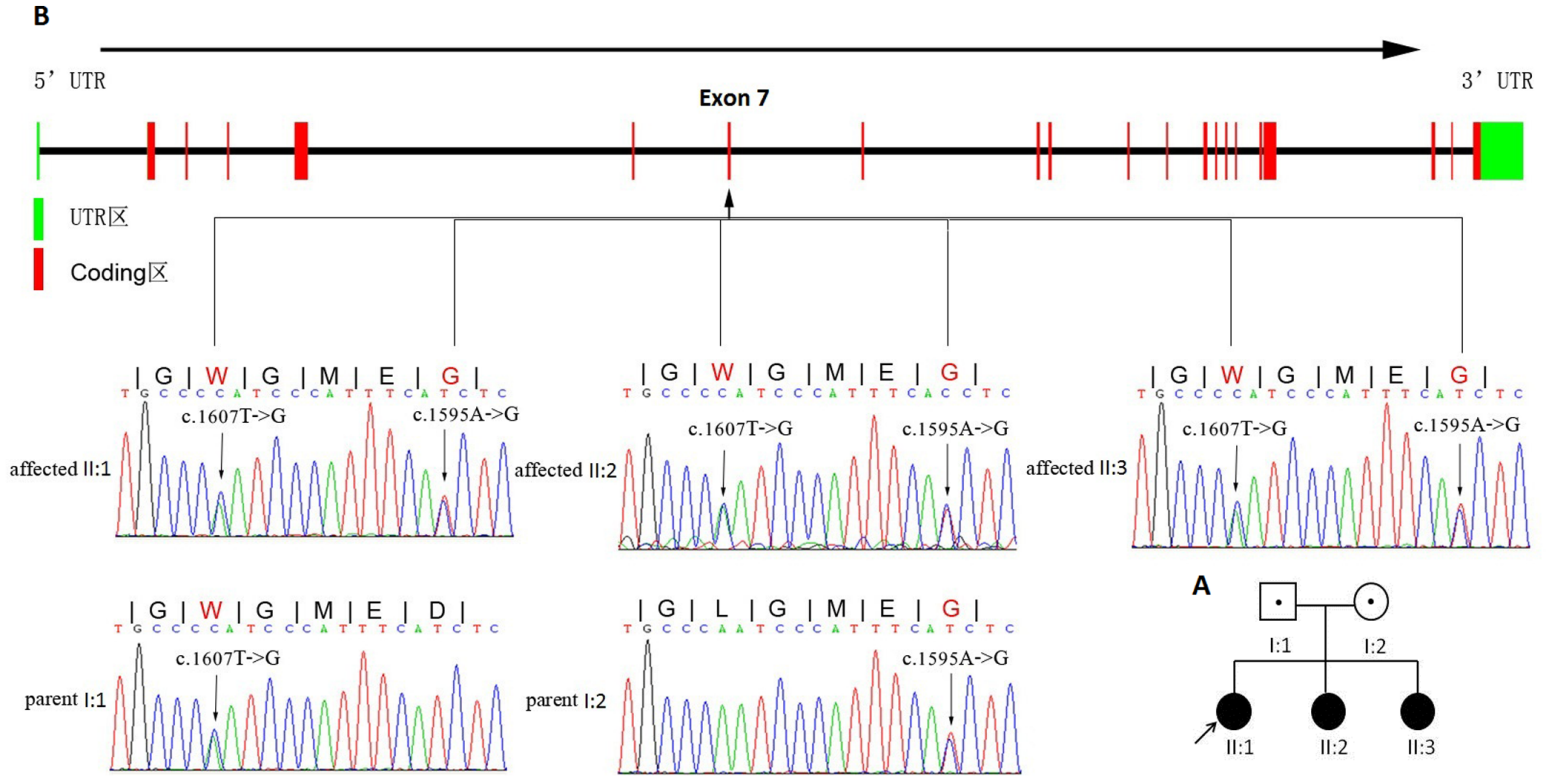
Compound heterozygous mutations and genomics structure of the exons of *ERCC6*. A) Pedigree of this family. Black symbols denote affected individuals, and open symbols denote unaffected individuals. B) The region of UTR (green) and coding (red) are listed in the *ERCC6* gene (upper panel). Sanger sequencing analysis of c.1607T->G (p.Leu536Trp) and c.1595A->G (p.Asp532Gly) mutations in five family members. The heterozygous missense mutation c.1607T->G was identified in proband’s father (Parent I:1), another heterozygous missense mutation c.1595A->G in proband’s mother (Parent I:2) and three affected sisters carried (Affected II:1, II:2, II:3) the compound heterozygous mutations.

**Figure 2:**
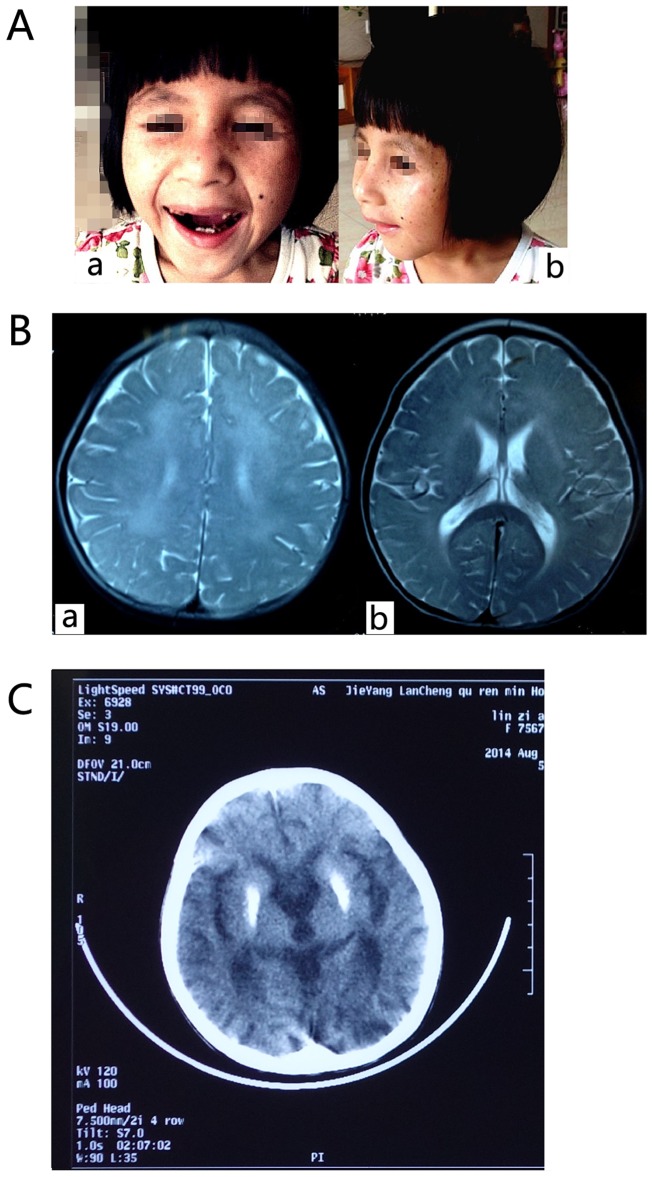
The face photo, skull MRI and CT scan of the proband. A) The face photo of the proband (a, the normal position; b, the lateral position). B) The skull MRI of the proband at 2 years old (a, the plain scanning; b, enhanced scanning). Hyperintensity was seen in centrum semiovale bilaterally, corona radiate, white matter of frontal and parietal lobe, anterior limb of internal capsule, external capsule in T2 weighted images. T2 weighted images show less low signal in the posterior limb of internal capsule. C) The brain CT scan shown that calcification on bilateral globus pallidus, cerebral atrophy (widened sulcus and cleft, enlarged superatentorial ventricle, narrowed gyrus).

The three affected individuals with Cockayne syndrome are sisters from a Chinese family (Fig. 1A). Their parents are nonconsanguineous and healthy. The three sisters were all born at full-term by vaginal delivery and had no abnormalities at birth. They had similar clinical features including postnatal growth failure, severe and progressive neurological deterioration, cachexia, and photosensitivity (Fig. 2A). The major clinical manifestations of the three sisters are summarized in Table 1. The results of laboratory tests for the proband (II1) were as follows: normal karyotype (46, XX); no subtelomeric duplications or deletions in any chromosomes, as detected by P036 and P070 Multiplex Ligation Dependent Probe Amplification (MLPA) kits (MRC-Holland, Netherlands); no microdeletion syndromes, as detected with a P245 MLPA kit (MRC-Holland, Netherlands); absence of clinically significant microdeletions and microduplications, as detected using a Array-CGH (Comparative genomic hybridization) Agilent 180K chip (Agilent, USA); and no inherited metabolic diseases, as detected by tandem mass spectrometric analysis. The cerebra MR imaging for the proband showed signs of hypomyelination (Fig. 2B). The brain CT scan showed that calcification on bilateral globus pallidus, cerebral atrophy (widened sulcus and cleft, enlarged superatentorial ventricle, narrowed gyrus) (Fig. 2C).

**Table 1:** Major clinical manifestations of the affected individuals.

Patients		II1	II2	II3
**Gestational age**		Full-term vaginal delivery	Full-term vaginal delivery	Full-term vaginal delivery
**Birth weight**		2.51 kg	2.50 kg	2.50 kg
**Age at clinic visit**		4 years and 8 months	3 years and 5 months	1 year and 9 months
**Gesell developmental scales**	**Adaptability**	developmental age equal to 15 months, developmental quotient 60	developmental age equal to 18 months, developmental quotient 44	developmental age equal to 12.5 months, developmental quotient 57
	**Gross motor**	developmental age equal to 13.8 months, developmental quotient 57	developmental age equal to 12.8 months, developmental quotient 32	developmental age equal to 11.8 months, developmental quotient 54
	**Fine motor**	developmental age equal to 14 months, developmental quotient 64	developmental age equal to 18 months, developmental quotient 44	developmental age equal to 14 months, developmental quotient 64
	**Language**	developmental age equal to 13 months, developmental quotient 56	developmental age equal to 15.6 months, developmental quotient 38	developmental age equal to 12.3 months, developmental quotient 56
	**Social and emotional development**	developmental age equal to 14 months, developmental quotient 62	developmental age equal to 20 months, developmental quotient 49	developmental age equal to 13.7 months, developmental quotient 62
**Physical examination**	**Height**	80 cm (<1th percentile)	76.5 cm (<1th percentile)	70 cm (<5th percentile)
	**Weight**	9 kg (<1th percentile)	7.8 kg (<1th percentile)	8.8 kg (<5th percentile)
	**head circumference**	44.1 cm (<3th percentile)	42 cm (<3th percentile)	42.5 cm (<3th percentile)
	**other**	pachylosis, deep-set eyes, beaked nose, loss of subcutaneous fat in the face, triangular face with sunburns, dental caries, enamel hypoplasia, malocclusion, muscular atrophy of legs, photosensitive skin rashes with a butterfly distribution in the face, walking unsteady.	pachylosis, deep-set eyes, beaked nose, loss of subcutaneous fat in the face, triangular face with sunburns, dental caries, enamel hypoplasia, malocclusion, muscular atrophy of legs, photosensitive skin rashes in the face.	pachylosis, no dental caries, muscular atrophy of legs.

### Exome capture and next-generation sequencing

Genomic DNA was extracted from peripheral blood using the QIAamp DNA Blood Mini Kit (Qiagen, Hilden, Germany) according to the manufacturer’s instructions. The genomic DNA of the three patients was then fragmented by Covaris S2 (Massachusetts, USA) to generate 200–300bp insert fragments. The paired-end libraries were prepared following Illumina library preparation the protocol. Exome capture was performed to collect the whole exons of the human genomic DNA using an Agilent SureSelect Human All Exon Plus kit (44 Mb Kit, Agilent Technology, Inc.). The exon-enriched DNA libraries were sequenced by 100 bp paired-end reads on a Hiseq2000 sequencer (Illumina, San Diego, California). Raw image files were processed by the Illumina Pipeline for base calling using default parameters.

### Reads mapping and variants analysis

Primary data came in fastq form after image analysis and base calling was conducted using the Illumina Pipeline. The data were filtered to generate “clean reads” by removing adapters and low quality reads (<Q20). Sequencing reads were mapped to the reference human genome version hg19 (200902 release, http://genome.ucsc.edu/) using the BWA (Burrows Wheeler Aligner) Multi-Vision software package. Variant analysis was performed using SOAPsnp software and Samtools for SNPs and indels, respectively. All SNPs were identified using the NCBI dbSNP137, HapMap, 1000 human genome dataset (20110521 release, http://www.1000genomes.org/) and a local database of 100 Chinese healthy adults.

### Sanger sequencing

Sanger sequencing was performed to confirm the two variants of the *ERCC6* gene found by exome sequencing. We amplified the target sites and the flanking sequences of the DNA template of each family member individually with specific primers designed using Primer5.0 (forward primer 5′-CTGCCCTACAGCTCCATT-3′ and reverse primer 5′-TCCACCATTTGCCATTTT-3′). PCR amplification was carried out in an ABI 9700 Thermal Cycler using standard conditions. PCR conditions were as follows: an initial denaturation step at 95°C for 5 min, followed by 94°C for 30 s, annealing at 55°C for 30 s, and an extension at 72°C for 30 s. A final extension of 72°C for 5 min was performed. After the amplification, products were purified using a Universal DNA Purification Kit (Tiangen) and directly sequenced on an ABI PRISM 3730 automated sequencer (Applied Biosystems). The sequencing results were compared with the annotated *ERCC6* gene reference sequence (NC_000010.10) to confirm the candidate nucleotide variants.

### Prediction analysis

We used dbNSFP (http://sites.google.com/site/jpopgen/dbNSFP) [7], which compiles prediction scores from SIFT, PolyPhen-2, LRT and MutationTaster, along with a conservation score (PhyloP), to predict the pathogenicity of the single-nucleotide substitution variants.

In addition, SWISS-Model Repository (http://swissmodel.expasy.org/repository/) [8] and PROSITE (http://www.expasy.org/prosite/) [9] were used to analyze the protein structure, conservation domain and functional domain.

## Results

### Exome Sequencing

We sequenced the exome of three affected individuals from the same family (Samples II:1, II:2 and II:3, Fig. 1A). We generated an average of 103.52 million reads of sequence with 128.87-fold average coverage for all affected individuals as paired-end, 90 bp reads. Approximately 99.22% of the reads passed the quality assessment and aligned to the human reference genome 19, and approximately 98.9% of the targeted bases were sufficiently covered to pass our thresholds for calling SNPs and InDels (Table S1).

According to the criteria mentioned above, we detected more than 80 thousand variants in each patient. After filtering for 364 candidate genes that are related to mental and psychomotor retardation, over 1600 variants were identified in each person. Next, we focused on nonsynonymous (NS) variants, splice acceptor and donor site mutations (SS) and coding indels (Indel) that were relatively more likely to be pathogenic mutations. Because CS is a rare disorder, the causative variants will occur at a very low frequency in the general population. Therefore, we compared the NS/SS/Indel variants against the dbSNP137, HapMap, 1000 human genome dataset and a local database of 100 Chinese healthy adults, and removed the shared SNPs. Given that these patients are related and that they are expected to share the causal variant for CS, we filtered all the detected variations in these patients against each other and found 7 shared variants. And due to the CS in this family is an autosomal recessive inherited disease, we found two SNPs are consistent with AR inherited mode (Table S2). The two SNPs (c.1595A>G, p.Asp532Gly and c.1607T>G, p.Leu536Trp) were compound heterozygous in all three sisters and were located in exon 7 of the *ERCC6* gene, which is related to the CS status.

### Variant validation by Sanger sequencing

To confirm the accuracy of the mutations identified by exome sequencing, PCR-based Sanger sequencing was performed to analyze the two missense mutations in the three affected sisters and unaffected parents. All three affected individuals carried these compound heterozygous mutations in *ERCC6* (c.1607T>G and c.1595A>G), and the unaffected parents carried one of them (Fig. 1B). The co-segregation analysis in this family indicated that the c.1607T>G (p.Leu536Trp) missense mutation was inherited from the unaffected father, and the other mutation (c.1595A>G, p.Asp532Gly) inherited from unaffected mother, consistent with the phenotype of this family. The results showed complete consistency between the exome sequencing and the Sanger sequencing, suggesting that exome sequencing provides high accuracy and can be applied to the analysis of clinical samples.

### Prediction analysis

**Figure 3:**
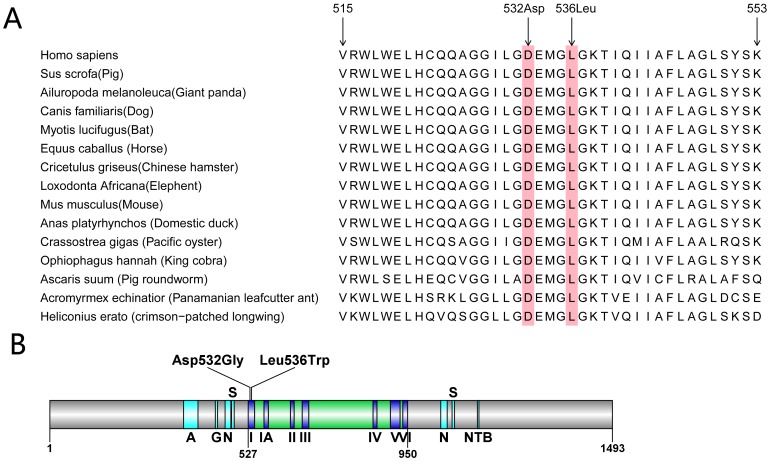
The conservation and domain structure of excision repair protein ERCC-6. A) Multiple sequence alignment of ERCC6 ATP-binding domain amino acids among homologous genes in mammals, reptile, nematode, oyster and insects both 532D and 536L heterozygous missense mutations are at a highly conserved position in *ERCC6*. B) The structure of CSB protein indicated that the two mutations D532G and L536W are located in motif I of the helicase domain. A, acidic amino acid stretch; G, glycine rich region; S, serine phosphorylation site preceded by a nuclear location signal; N, nucleotide binding fold; NTB, nucleotide binding fold; I, IA, and II-VI refer to the corresponding helicase motifs.

The *ERCC6* gene is located at 10q11.23 and encodes a 1493aa protein called excision repair protein (ERCC6). It is also named ATP-dependent helicase ERCC6 or Cockayne syndrome protein (CSB protein). CSB protein contains an ATP-binding domain from residue 519 to residue 695 [10]. This ATP-binding domain is highly conserved in mammals, reptile, nematode, oyster and insects compared with *Sus scrofa, Ailuropoda melanoleuca, Canis familiaris, Myotis lucifugus, Equus caballus, Cricetulus griseus, Loxodonta Africana, Mus musculus, Anas platyrhynchos, Crassostrea gigas, Ophiophagus hannah, Ascaris suum, Acromyrmex echinatior* and *Heliconius erato* (Fig. 3A). The structure of the CSB protein is shown in Fig. 3B. It contains two essential components: ATPase and Ubiquitin binding domain [11]. The two mutations D532G and L536W are located immediately in motif I of the ATPase domain (Fig. 3B), and have been characterized as “Damaging,” “Probably Damaging,” “Disease-causing,” “Conserved” and “NA” using SIFT, Polyphen2, MutationTaster, PhyloP and LRT, respectively. In addition, these two mutations were detected in another 105 Chinese individuals (63 healthy controls and 42 patients of other disease), but none of them had both mutations. All of these results indicate that those two mutations are the most probable cause of Cockayne’s syndrome.

## Discussion

Cockayne’s syndrome (CS) is a rare autosomal recessive disorder that results in postnatal growth failure, microcephaly, sensorial impairment, cutaneous photosensitivity, recognizable facial appearance with deep sunken eyes and progressive neurologic dysfunction, most symptoms appear and worsen with time. This disorder is classified as a segmental progeria that arises due to a mutation in *ERCC8* and *ERCC6*. In previous studies, short insertions and deletions, nonsense mutations, splice mutations, missense mutations and promoter mutation have all been implicated in Cockayne’s syndrome, and among the 84 reported cases up to 2009, approximate 65% had mutations in the *ERCC6* [5], [12]. A classic Cockayne’s syndrome case has been reported carrying a novel homozygous nonsense mutation c.1387C>T/Q463X in the *ERCC6* gene [13]. Another report identified two novel *ERCC6* mutations (a splice-site mutation, c.2709+1G>T, in intron 14 and a short deletion in exon 5 (c.1293_1320del) in an Amish individual with Cockayne’s syndrome [14].

In the present study, we performed exome sequencing for genetic testing of three sisters with postnatal growth failure and neurological impairment using the Hiseq2000 platform. After exome sequencing analysis and criteria filtering, we identified two missense mutations in the *ERCC6* gene in the three affected sisters. The c.1595A>G missense mutation results in the substitution of aspartic acid (D) for glycine (G) at residue 532,which has never been reported. The c.1607T>G missense mutation results in the substitution of leucine (L) for tryptophan (W) at residue 536 that had been recorded but no details [15]. The Sanger sequencing and co-segregation analysis in this family indicated that the compound heterozygous c.1595A>G and c.1607T>G mutations in the three affected sisters were inherited from their unaffected father and mother, respectively, consistent with the phenotypes of these family members. Additionally, the c.1595A>G missense mutation and the c.1607T>G missense mutation are predicted to be “Damaging” and “Probably damaging”, respectively, by SIFT and PolyPhen-2, and both are located in the highly conserved motif I of ATP-binding helicase.

The *ERCC6* gene encompasses 21 exons and yields an 8993 bp transcript that encodes the 1493aa CSB protein. It is a component of the nucleotide excision repair (NER) pathway, a complex system that eliminates a broad spectrum of structural DNA lesions, including ultraviolet (UV)-induced cyclobutane pyrimidine dimers, bulky chemical adducts, and DNA cross-links [16], [17]. The cellular roles played by the CSB protein in repair, transcription, chromatin remodeling and apoptosis may be mediated by distinct functional domains of the protein [18]. The conserved helicase ATPase has been shown to be an essential functional domain of the CSB protein in responding to UV-induced DNA damage in previous studies [19]–[21]. Furthermore, other studies have suggested that the recruitment of CSB to lesion-stalled transcription and chromatin remodeling is an ATP-dependent process [22]. Evidence indicates that the integrity of the motif I and II are important for CSB function. A point mutation in ATPase motif I of CSB rendered the protein totally defective in ATP hydrolysis, but it was still able to partially rescue the delay in RNA synthesis recovery after exposure to UV light when microinjected into an immortalized human CS-B fibroblast cell line [23]. A point mutation in motif II of the ATPase domain completely abolished the ability of CSB to function in survival, RNA synthesis recovery, gene-specific repair and apoptosis upon UV irradiation in hamster and human cells [24], [25]. And other study demonstrated the point mutations in ATPase motif Ia, III, V and VI also abolished the genetic function of the CSB protein in survival, RNA synthesis recovery and apoptosis after UV treatment [18]. Therefore, the two missense mutations, D532G and L536W, located in the motif I of ATP-Binding domain loss of ATP hydrolysis and then fail to repair the DNA damage which maybe the pathogenic reason of the three sisters.

In summary, we have described three Chinese sisters with Cockayne’s syndrome. Exome sequencing was performed, identifying two missense mutations p.Asp532Gly that is never been reported andp.Leu536Trp in the *ERCC6* gene. The results not only enrich the known mutation spectrum of this DNA repair disorder, but also indicate that whole exome sequencing will be a powerful tool for discovering the disease causing mutations in clinical diagnosis.

## Supporting Information

Table S1 **Summary of exome sequencing data.**
(DOCX)

Table S2 **Candidate genetic variants identified.**
(DOCX)
