# Supplementary material for: Identification of Two Missense Mutations of ERCC6 in Three Chinese Sisters with Cockayne Syndrome by Whole Exome Sequencing
Source: PLoS One. 2014 Dec 2;9(12):e113914. doi: 10.1371/journal.pone.0113914 (PMC4252064; doi:10.1371/journal.pone.0113914)
Supplement: Table S1 — Summary of exome sequencing data. (DOCX) [file pone.0113914.s001.docx]

**Table S1** Summary of exome sequencing data

| **Sample** | **Bases (Gbp)** | **Map Bases Rate (%)** | **Target Region Map Bases (Gbp)** | **Target Region Map Bases Rate (%)** | **Coverage (%)** | **Mean Depth** |
| --- | --- | --- | --- | --- | --- | --- |
| II:1 | 109.35 | 99.20% | 86.00 | 78.65 | 98.74 | 134.27 |
| II:2 | 101.59 | 99.20% | 80.83 | 79.56 | 99.03 | 125.83 |
| II:3 | 99.62 | 99.25% | 81.00 | 81.31 | 98.92 | 126.52 |
| **Average** | **103.52** | **99.22%** | **82.61** | **79.84** | **98.90** | **128.87** |
