# Supplementary material for: Identification of Two Missense Mutations of ERCC6 in Three Chinese Sisters with Cockayne Syndrome by Whole Exome Sequencing
Source: PLoS One. 2014 Dec 2;9(12):e113914. doi: 10.1371/journal.pone.0113914 (PMC4252064; doi:10.1371/journal.pone.0113914)
Supplement: Table S2 — Candidate genetic variants identified. (DOCX) [file pone.0113914.s002.docx]

**Table S2** Candidate genetic variants identified

| **Filter** | **II:1** | **II:2** | **II:3** |
| --- | --- | --- | --- |
| All SNPs/InDels | 85650 | 82482 | 80930 |
| In candidate genes | 1669 | 1677 | 1602 |
| NS/SS/Indel | 195 | 193 | 201 |
| Not in dbSNP137, HapMap, 1000 human genome dataset and local database of 100 Chinese healthy adults | 16 | 20 | 17 |
| II:1,II:2 and II:3 shared | 7 | | |
| AR inherited mode | 2 | | |
